# Theoretical Investigation of the Mechanism by which A Gain-of-Function Mutation of the TRPM4 Channel Causes Conduction Block

**DOI:** 10.3390/ijms22168513

**Published:** 2021-08-07

**Authors:** Yaopeng Hu, Qin Li, Yanghua Shen, Takayuki Fujita, Xin Zhu, Ryuji Inoue

**Affiliations:** 1 Department of Physiology, Fukuoka University School of Medicine, Fukuoka 814-0180, Japan; fujitaka@fukuoka-u.ac.jp; 2 Biomedical Information Engineering Lab, Division of Information Systems, the University of Aizu, Aizu-Wakamatsu 965-8580, Japan; aasynch@gmail.com (Q.L.); envy518@gmail.com (Y.S.); zhuxin@u-aizu.ac.jp (X.Z.)

**Keywords:** transient receptor potential melastatin subfamily, inherent cardiac arrhythmia, conduction block, gating analysis, numerical simulation

## Abstract

In the heart, TRPM4 is most abundantly distributed in the conduction system. Previously, a single mutation, ‘E7K’, was identified in its distal N-terminus to cause conduction disorder because of enhanced cell-surface expression. It remains, however, unclear how this expression increase leads to conduction failure rather than abnormally enhanced cardiac excitability. To address this issue theoretically, we mathematically formulated the gating kinetics of the E7K-mutant TRPM4 channel by a combined use of voltage jump analysis and ionomycin-perforated cell-attached recording technique and incorporated the resultant rate constants of opening and closing into a human Purkinje fiber single-cell action potential (AP) model (Trovato model) to perform 1D-cable simulations. The results from TRPM4 expressing HEK293 cells showed that as compared with the wild-type, the open state is much preferred in the E7K mutant with increased voltage-and Ca^2+^-sensitivities. These theoretical predictions were confirmed by power spectrum and single channel analyses of expressed wild-type and E7K-mutant TRPM4 channels. In our modified Trovato model, the facilitated opening of the E7K mutant channel markedly prolonged AP duration with concomitant depolarizing shifts of the resting membrane potential in a manner dependent on the channel density (or maximal activity). This was, however, little evident in the wild-type TRPM4 channel. Moreover, 1D-cable simulations with the modified Trovato model revealed that increasing the density of E7K (but not of wild-type) TRPM4 channels progressively reduced AP conduction velocity eventually culminating in complete conduction block. These results clearly suggest the brady-arrhythmogenicity of the E7K mutant channel which likely results from its pathologically enhanced activity.

## 1. Introduction

TRPM4 is a melastatin subfamily member of the transient receptor potential (TRP) superfamily and acts as a plasmalemmal route selective for Na^+^ influx and K^+^ efflux. In physiological settings, the TRPM4 channel is directly activated by intracellular Ca^2+^ elevation upon transmembrane and intracellular Ca^2+^ mobilizations. The distribution of this channel protein is ubiquitous across the whole body including both excitable (neurons, muscles) and non-excitable (secretory glands, blood cells, etc.) tissues. Thus, the TRPM4 channel is regarded as the most likely molecular identification of a broad class of Ca^2+^-activated nonselective cation channels [1]. Because of its predominant Na^+^ permeability, major consequences of TRPM4 channel activation are thought to be two-fold, i.e., membrane depolarization and intracellular Na^+^ loading. Generally, in non-excitable cells, the depolarizing effect of TRPM4 channel activation reduces non-voltage-gated Ca^2+^ influxes via decreasing the Ca^2+^ driving force. For example, in Jurkat T lymphocytes, the activation of TRPM4 channels was found to attenuate store-operated Ca^2+^ influx, thereby inhibiting interleukin-2 synthesis [2]. In excitable cells, membrane depolarization resulting from TRPM4 channel activation secondarily elicits action potentials (APs) via activation of voltage-dependent Na^+^ and/or Ca^2+^ channels to facilitate neurotransmission and cause muscle contraction. In the heart, TRPM4 has been suggested to play pleiotropic roles; while it acutely modulates inotropic, chronotropic and dromotropic properties of the heart in both positive and negative fashions [3,4,5,6,7], it also chronically modifies remodeling processes such as physiological and pathological hypertrophies via regulation of non-voltage-gated Ca^2+^ influxes [8,9].

In the past decades, genetic linkage analyses and subsequent cohort studies have identified dozens of *trpm4* gene mutations associated with conduction disorders such as progressive familial heart block type I, isolated cardiac conduction disorder, atrio-ventricular block, right-bundle branch block, and Brugada syndrome. However, somewhat contra-intuitively, in vitro assays show that many of these mutations exhibited increased rather than decreased TRPM4 channel expression/activity [10]. For instance, the first identified TRPM4 mutation (E7K) was reported to increase cell-surface expression of TRPM4 channel protein (about twice) due to impaired SUMOylation without noticeable changes in its gating kinetics [11]. This might cause the depolarizing shift of resting membrane potential which would in turn facilitate the inactivation of voltage-dependent Na^+^ channel and decelerate AP propagation [12], but there is little evidence to validate this possibility. Furthermore, in our numerical model simulations, simple doubling of the maximal wild-type TRPM4 channel activity produced only marginal changes in the resting membrane potential and the shape of AP [13]. In addition, our recent study disclosed the functional abnormality of E7K mutation that unusually strengthens the interaction of TRPM4 channel activity with endogenous PIP_2_ thereby increasing the risk of generating triggered activities [14]. These facts raise the question of whether additional functional changes may also be involved in the pathogenesis of E7K-associated conduction disorders.

In the present study, to pursue this possibility, we adopted the following experimental approaches. First, we rigorously evaluated the gating kinetics of the ‘E7K’ mutant TRPM4 channel by use of ionomycin-perforated cell-attached (Iono-C/A) recording technique that allowed to stably record desensitization/rundown-prone TRPM4 channel activities [13]. Kinetic data obtained from these experiments were then mathematically formulated as the rate constants of opening and closing, and they were incorporated into the most updated single-cell AP model reflecting the unique electrophysiology and intracellular Ca^2+^ dynamics of human cardiac Purkinje fiber [15]. Finally, this modified model was used to perform 1D-cable simulations to investigate the arrhythmogenic impact of the E7K mutation. The results indicate that the E7K mutation greatly increases the sensitivity of TRPM4 channels to voltage and intracellular Ca^2+^ concentration ([Ca^2+^]_i_) to favor a longer sojourn in the open state and this property renders this mutant channel contributive to conduction failure.

## 2. Results

### 2.1. Gating Analysis Reveals That E7K Mutation Facilitates TRPM4 Channel Opening

With normal recording variants of patch clamp technique, the activity of expressed TRPM4 declined quickly because of rapid Ca^2+^-dependent desensitization and rundown [13,16]. To circumvent this, we developed a new recording technique dubbed ‘ionomycin-perforated cell-attached (Iono-C/A)’ recording [13]. By using this recording procedure, it was possible to stably record TRPM4-mediated currents up to about 10 min upon repeated exposure to different concentrations of Ca^2+^ in the bath.

**Figure 1:**
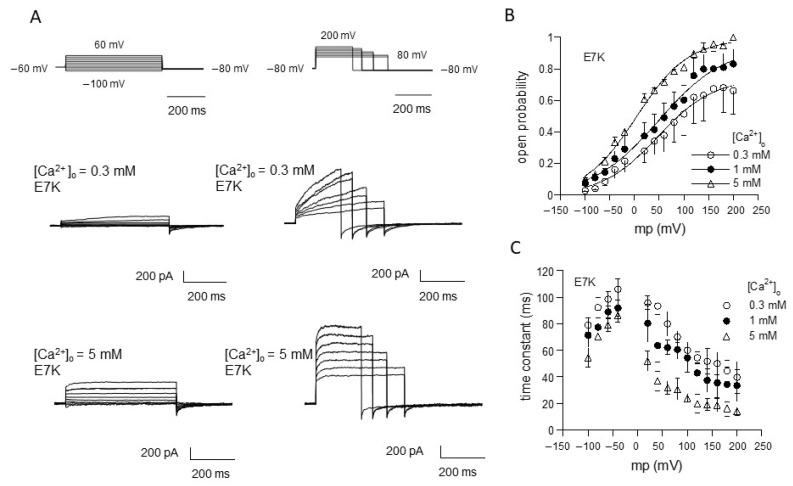
Evaluation of E7K-TRPM4 channel gating by voltage-jump experiments. (**A**) actual traces of E7K-mutant TRPM4 channel currents responding to a set of voltage pulses from−100 to 60 mV (left row) and 80 to 200 mV (right row) by 20 mV steps. In each row, top panels show voltage pulses, while middle and bottom panels show corresponding TRPM4-mediated currents at 0.3 and 5 mM Ca^2+^ in the bath. The TRPM4-mediated currents were recorded by Ion-C/A recording where 5 μM ionomycin-containing Ca^2+^-free solution was applied onto the cell immediately after giga-seal formation to permeabilize the cell membrane, which was then switched to solutions with various concentrations of extracellular Ca^2+^ ([Ca^2+^]_o_) to activate the current. All these agents were applied through a fast solution device ‘Y-tube’. It should be noted that the level of a rise in intracellular Ca^2+^ concentration ([Ca^2+^]_i_) corresponding to a given [Ca^2+^]_o_, which was separately determined by Ca^2+^ imaging experiments, was almost stable and did not involve Ca^2+^ release from internal stores [13]. (**B**,**C**) the relationship of open probability (P_o_) versus membrane potential (**B**) and that of time constant (τ) of activation/deactivation time courses during voltage pulses (**C**). Different symbols and bars in the figure denote mean ±S.E.M. from five independent experiments. Smooth curves in A are drawn according to the best fits of data points (normalized to the maximum) by the Boltzmann equation: 1/[1 + exp(−(V_m_ − V_0.5_)/s), where V_m_, V_0.5_ and s denote membrane potential, half-activation voltage and slope factor, respectively. The values of these parameters are 47.542, 48.825 and 3.697 mV, and 56.582, 67.327 and 52.844 mV for 0.3, 1 and 5 mM [Ca^2+^]_o_, respectively.

We applied this procedure to evaluate the gating behavior of the TRPM4 channel carrying the ‘E7K’ mutation by means of voltage-jump protocols (Figure 1). In response to step depolarizations, TRPM4-mediated currents (induced by Ca^2+^) showed time-dependent increases in the amplitude. On the contrary, repolarizations back to more negative potentials deactivated the currents (see the tail currents just after the end of depolarizing pulses in Figure 1A). Figure 1B,C, respectively, show the averaged values of variables for steady-state activation [open probability (P_o_)] and activation/deactivation time courses (time constant (τ) after normalization with respect to three different concentrations of Ca^2+^ in the bath ([Ca^2+^]_o_; note that these are later converted to [Ca^2+^]_i_ values; for details, see Figure 1 legend). Similar to the wild-type TRPM4 channel [13,17], the relationships of P_o_ and τ against the membrane potential (V_m_) for E7K show clear voltage dependencies, and these are modified by Ca^2+^. Boltzmann fitting of the P_o_-V_m_ relationships for the E7K mutant demonstrate that increasing [Ca^2+^]_o_ shifts the voltage-dependent activation curve toward more negative V_m_. Furthermore, the values of the half-activation voltage (V_0.5_), which is an indication for the dynamic V_m_ range of a channel being open, are 47.5, 48.8 and 3.7 mV for [Ca^2+^]_o_ of 0.3,1 and 5 mM, respectively (these values are 54.7, 80.4 and 38.9 mV for the wild-type TRPM4 [13]). This suggests that the voltage dependency of the E7K mutant may be more susceptible to Ca^2+^ than the wild-type TRPM4 channel [13].

**Figure 2:**
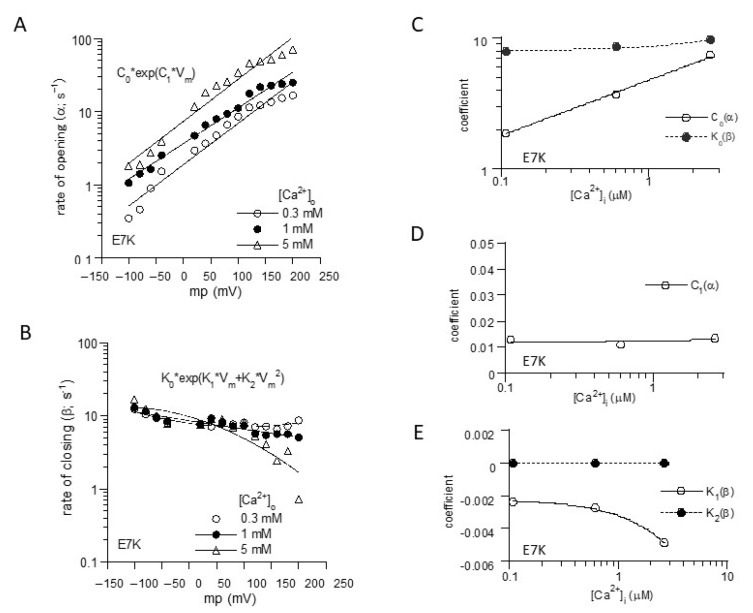
Mathematical formulation of voltage-and Ca^2+^-dependent gating of E7K-TRPM4 channels. (**A**) and (**B**); The voltage-dependencies of α and β were empirically fitted by the exponentials with the first and second-order V_m_ polynominals, respectively. (**C**–**E**); the coefficients for each polynominal (C_0_, C_1_; K_0_–K_2_) obtained in A and B were further empirically fitted with respect to intracellular Ca^2+^ concentration ([Ca^2+^]_i_) by linear or exponential fittings. [Ca^2+^]_i_ was determined by separately preformed [Ca^2+^]_i_ measurements with the fura-2 digital fluorescence imaging [13].

To more unequivocally elucidate the altered gating by the ‘E7K’ mutation, we calculated the rate constants of opening (α) and closing (β) from the voltage and Ca^2+^ dependencies of P_o_-V_m_ and τ-V_m_ relationships (Figure 1B,C), based on the two-state C-O transition scheme (Figure 2). From these, the final mathematical expressions for α and β were obtained as the functions of V_m_ and [Ca^2+^]_i_:

(1) $$\alpha \left(V,\left[Ca\right]\right)=4.767\cdot {\left[Ca\right]}^{0.42534}\cdot \exp\left[\left(0.012121+0.000000034609\cdot \left[Ca\right]\right)\cdot V\right]$$

$$\left(\mathrm{in}s^{-1};[\mathrm{Ca}]\mathrm{in}\mu M,V\mathrm{in}\mathrm{mV}\right)$$

(2) $$\beta \left(V,\left[Ca\right]\right)=0.37975\cdot \exp[\left(3.046+0.07528\cdot \left[Ca\right]\right)+\left(-0.0022098-0.0010278\cdot \left[Ca\right]\right)\cdot V\;+\left(0.000011-0.0000118\cdot \left[Ca\right]\right)\cdot V^{2}]$$

$$\left(\mathrm{in}s^{-1};[\mathrm{Ca}]\mathrm{in}\mu M,V\mathrm{in}\mathrm{mV}\right)$$

**Figure 3:**
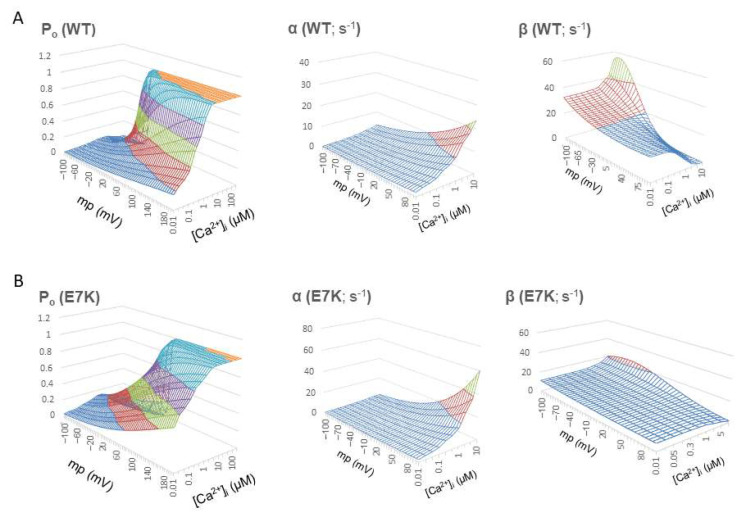
Voltage- and Ca^2+^-dependent profiles of Po, α and β. P_o_, α and β were recalculated over wide ranges of V_m_ and [Ca^2+^]_i_ by using the mathematical expressions of α and β shown above. For wild-type TRPM4, similar mathematical expressions for α and β which had been obtained from wild-type TRPM4 expressing HEK293 cells [13] were used to calculate P_o_, α and β. A and B; surface plots of P_o_, α and β (from left to right) for wild-type TRPM4 channel against two variables V_m_ and [Ca^2+^]_i_ (**A**) and those for E7K-mutant TRPM4 channel (**B**). Note that P_o_ contour is less steep for E7K mutant with increased voltage-dependence of α and prominently decreased voltage and Ca^2+^ dependency of β which stay low over the whole V_m_ and [Ca^2+^]_i_ ranges. These results indicate a preferred sojourn in the open state of E7K channel due to accelerated C-O transition and greatly suppressed O-C transition.

Reconstructed relationships of P_o_, α and β versus V_m_ and [Ca^2+^]_i_ indicate that the E7K mutation renders the channel more readily open at more negative membrane potentials and at lower [Ca^2+^]_i_ by enhancing the voltage and Ca^2+^ dependencies of α and decreasing those of β. In other words, this mutation accelerates the closed-to-open state (C-O) transition and simultaneously decelerates the reverse (O-C) transition of TRPM4 channels via facilitated voltage-dependent gating and increased Ca^2+^-sensitivity (Figure 3).

**Figure 4:**
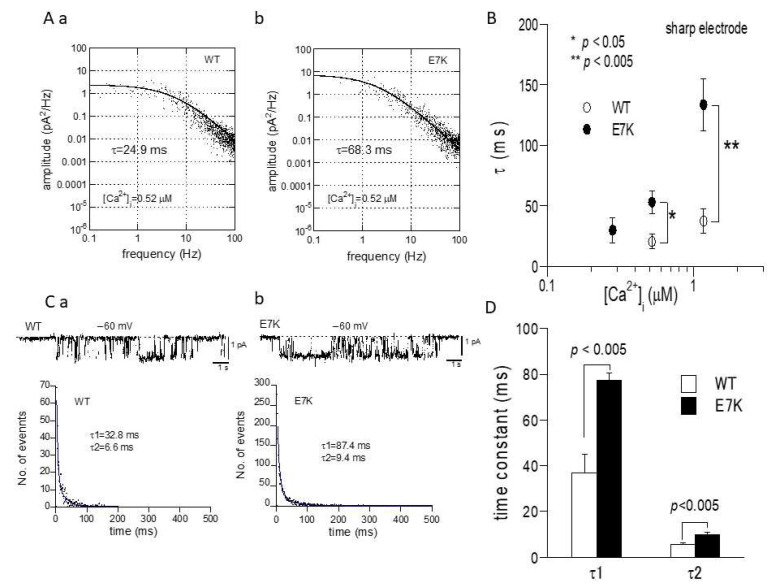
Noise spectrum and single channel analyses of E7K channel gating. (**A**) representative spectral density plots of wild-type (a) and E7K-TRPM4 (b) whole-currents at −60 mV at 0.52 μM [Ca^2+^]_i_. Each plot is fitted by the equation: s(f) = s(0)/[1 + (f/f_c_)^2^] (see the Methods). The value of time constant (τ) is calculated according to the relationship: τ = 1/2πf_c_. The τ value would provide a reasonable estimate for the open life-time, provided that the rate of closing is much larger than that of opening, i.e., near the resting membrane potential (see Figure 3). To minimize the desensitization/rundown of currents, sharp patch electrodes were used to infuse desired [Ca^2+^]_i_ into the cell. (**B**) summary of spectrum analysis such as shown in A. Open and filled circles and bars indicate the means ± S.E.M.s of τ at 0.28, 0.52 and 1.17 μM [Ca^2+^]_i_ (*n* = 4–8). Each condition was tested independently. Only the data at the same [Ca^2+^]_i_ was compared by unpaired *t*-test. *, **: *p* < 0.05 and 0.005. (**C**) representative traces of single channel activities at −60 mV (upper) and their respective dwell-time histograms (lower) for wild-type (a) and E7K- TRPM4 (b) channels. Their dwell-time histograms (shown in dots) are fitted by a sum of two exponentials with fast and slow time constants of τ1 and τ2. (**D**) averaged values of τ1 and τ2 with S.E.M bars for wild-type and E7K-mutant TRPM4 channels. *n* = 7. *p* values are the results of unpaired *t*-tests.

**Figure 5:**
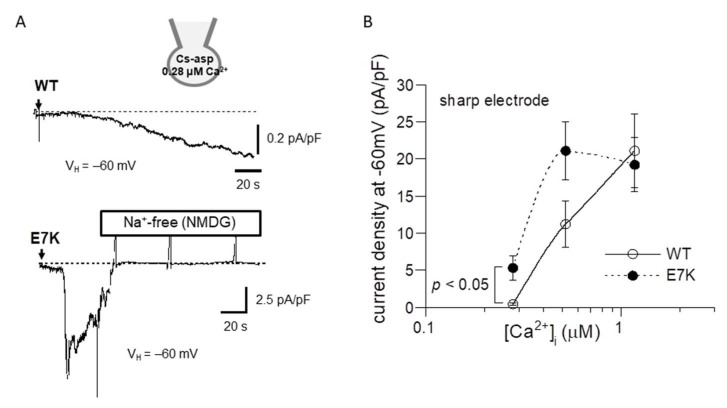
Ca^2+^-dependent activation curves for wild-type and E7K-TRPM4 currents. Internal solutions containing three different [Ca^2+^]_i_ were introduced via sharp thin patch electrodes into the cell by breaking the giga-sealed membrane at a holding potential of −60 mV. (**A**) representative traces of 0.52 μM [Ca^2+^]_i_ -induced currents from HEK293 cells expressing either wild-type (upper) or E7K-mutant (lower) TRPM4 channels. The arrows indicate the timepoints of ‘break-in’. Vertical positive deflections in the figure show the membrane currents evoked by ramp voltages. (**B**) the relationships between the TRPM4 current amplitude (at −60 mV) and intracellularly perfused [Ca^2+^]_i_. Open and filled circles represent wild-type and E7K-mutant TRPM4 channels. *n* = 4–12. Each condition was tested independently. Only the data at the same [Ca^2+^]_i_ was compared by unpaired *t*-test. *, **: *p* < 0.05 and 0.005.

To confirm this prediction by patch clamp experiments, we next performed the power spectrum analysis of whole-cell TRPM4 currents induced by direct Ca^2+^ infusion via a sharp electrode [13] and the dwell-time analysis of single TRPM4 channel activities. As demonstrated and summarized in Figure 4, the open life-time of the E7K-mutant was significantly longer than that of the wild-type in single-channel analysis, which appears to match up with the estimation from the power spectrum analysis. In addition, the relationship between the intracellularly perfused [Ca^2+^]_i_ and the functional density of the resultant whole-cell E7K-TRPM4 current was significantly leftward-shifted with most robust activation near the resting [Ca^2+^]_i_ level (0.28 μM), as compared with that of the wild-type TRPM4 current (Figure 5). These results are consistent with the theoretical prediction of enhanced Ca^2+^ susceptibility by the E7K mutation.

### 2.2. Numerical Simulations Show That Preferred Opening by E7K Mutation Causes Conduction Block

**Figure 6:**
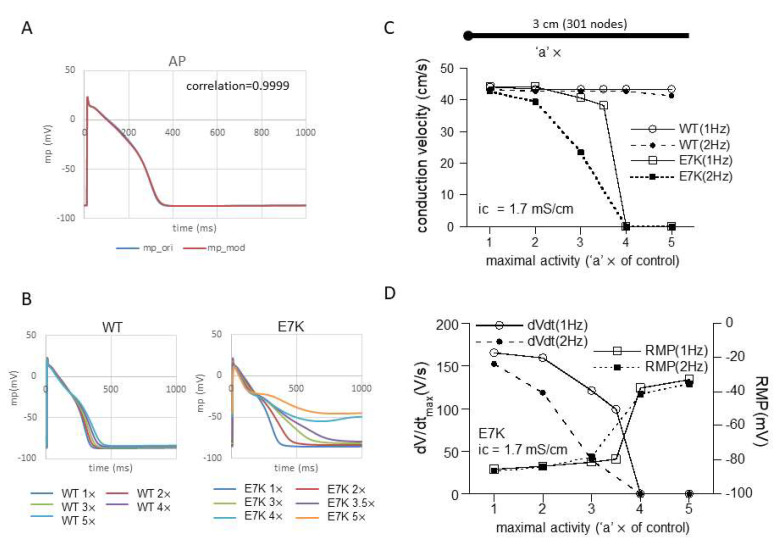
E7K mutation causes conduction block. (**A**) modified human Purkinje fiber Trovato2020 model (for details, see the Methods) was employed to simulate action potential (AP) and conduction velocity (CV). A; adaption of TRPM4 gating kinetics to the original Trovato model. (**B**) single-cell AP simulations by modified Trovato model incorporated with the gating kinetics of either wild-type (left) or E7K-mutant (right). The results are overlaid for each. (**C**) relationships between conduction velocity (CV) and channel density (or maximal activity) with intracellular conductivity of 1.7 mS/cm at 1 or 2 Hz pacing. The channel density is presented as a multiple (‘a’ ×) of control (i.e., 7.02 × 10^−8^ Litre/farad/ms) for wild-type and E7K-TRPM4 channels. The horizontal bar on the top panel in C denotes a 3 cm-long cable (stimulating point of 0–0.05 cm is shown as a large filled circle on the left). To avoid boundary effects, CV was calculated between 0.2 and 2.8 cm. (**D**) relationships of maximal upstroke slope of propagated AP (dV/dt_max_; measured at 2.8 cm) or resting membrane potential (RMP) against TRPM4 channel density which is shown as the fold increase (‘a’ ×) as in (**C**).

Intuitively, enhanced voltage-and Ca^2+^-dependency, in particular in their low physiological ranges, may imply more excitatory influences of the E7K mutation on both AP generation and conduction of the heart. In fact, computer simulation of single-cell AP shows that regardless of numerical models employed, the facilitated opening of the E7K-mutant TRPM4 channel much more readily induces AP prolongation as its cell-membrane density increases, compared with the wild-type TRPM4 channel (Figure 6B and Figure S1). Furthermore, when the prolongation is prominent, small oscillatory depolarizations which resemble early after-depolarizations (EADs) appear on the repolarizing phase of AP (Figure 6B).

**Figure 7:**
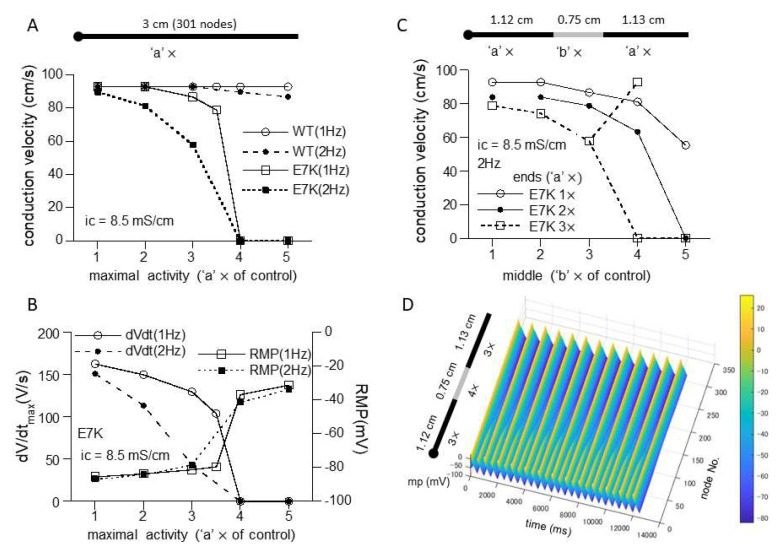
Heterogeneous expression of E7K causes partial conduction block. (**A**,**B**) relationships of CV (A), dV/dt_max_ (B) or RMP (B) against the density of wild-type or E7K mutant TRPM4 channels with the intracellular conductivity of 8.5 mS/cm. Definitions of the ordinate and abscissa are the same as in Figure 6C,D. (**C**) CV-channel density relationship for E7K-mutant TRPM4 channel under spatial heterogenous conditions consisting of two different densities at the middle (‘b’ ×) and both ends (‘a’ ×). For better visualization, only the cases for 1× to 3× with intracellular conductivity of 8.5 mS/cm and 2 Hz-pacing are shown. (**D**) spatiotemporal profile (surface plot) of partial conduction block (2:1) observed for the case of open squares in C in which the densities in the middle and ends are set to 4× and 3×, respectively. The last 25 out of 50 stimulations are shown.

However, when a 1D-cable model was employed to compute AP conduction, different consequences of the mutation were revealed. Figure 6C and Figure 7A recapitulate the influence of increased maximal channel activity on the conduction velocity (CV) along a 3 cm-long cable. Because TRPM4 is reported to be expressed most abundantly in the conduction system, we employed the most updated human single-cell AP model for Purkinje fiber [15]. The results of simulation clearly indicate that while CV is only slightly affected by 1- to 5-fold increases in wild-type TRPM4 channel density, the same degrees of the density increases cause progressive reductions in CV for the E7K mutant eventually culminating in complete conduction block. The extent of CV reduction appears strengthened by a faster pacing (2 Hz) but little dependent on the intracellular conductivity. The reduction in CV in the E7K mutant channel is proportionate to the decrease in the maximal slope of propagated AP upstroke (dV/dt_max_) and appears correlated with the depolarizing shift of the resting membrane potential (RMP) (Figure 6C,D and Figure 7A,B). Interestingly, introducing heterogeneity in the channel density along the cable (see also in [18]) created more complex patterns of conduction block such as 2:1 or 3:1 conductions which were split into non-conductive and full- or sub-conductive states (Figure 7C). These results strongly suggest that the impact of facilitated opening by the E7K mutation would manifest differentially at the single-cell and structurally higher levels.

## 3. Discussion

In the present study, we theoretically investigated the gating kinetics of a gain-of-function mutant of TRPM4 ‘E7K’ to show its pathophysiological significance in conduction disorder by means of gating analysis and numerical simulation. For this purpose, we adopted the Iono-C/A recording technique that allowed us to stably record TRPM4-mediated currents by minimizing rapid Ca^2+^ desensitization and rundown [13,16]. The results of gating analysis indicated that both voltage and Ca^2+^ dependencies of opening and closing rate constants are markedly affected by the E7K mutation so as to increase the open probability of TRPM4 channels in particular near the resting membrane potential and resting [Ca^2+^]_i_ (Figure 3). Furthermore, this theoretical prediction was confirmed by the power spectrum and single channel analyses, both of which showed prominent prolongation of open-life times of the E7K mutant channel (Figure 4 and Figure 5).

Incorporating the altered gating of the E7K mutant into a single-cell Purkinje fiber model demonstrated that increasing the channel density 1-to 5-fold produces density-dependent AP prolongation which ultimately converges to partially depolarized levels. In contrast, the same degrees of increases only slightly affect the shape and duration of AP as well as the resting membrane potential (RMP) in the wild-type TRPM4 channel (Figure 6B). These disparate effects on AP and RMP are clearly reflected in AP conduction. While the speed of AP conduction along the 1D-cable stays almost constant in the wild-type, that of the E7K mutant progressively diminishes as its density (maximal activity) increases (Figure 6C and Figure 7A). The slowing of conduction (CV reduction) is well proportionate to the reduction in dV/dt_max_ (Figure 6D and Figure 7B), the measure for the magnitude of voltage-dependent Na channel (Na_v_) current primarily contributing to AP upstroke [19]. Although there is considerable complexity due to local loading effects and structural discontinuities at tissue levels [20], this finding is consistent with the general idea that the velocity of AP propagation is correlated with the magnitude of a local Na_v_ current flow originating from the excited region. Moreover, the decreases in dV/dt_max_ and CV also coincide with the depolarizing shifts of membrane potential prior to AP upstroke, i.e., RMP. The level of RMP is crucial to determine Na_v_ availability just before AP generation, and in fact, the extent of the observed RMP shift reasonably accounts for the decrease in Na_v_ availability at AP upstroke which is estimated from its voltage-dependent inactivation curve (not shown). In aggregate, these results provide compelling evidence for the previous speculation [12] that excessive E7K-TRPM4 activities at resting conditions would facilitate Na_v_ inactivation during diastole thereby slowing the generation and subsequent propagation of AP. However, it should be noted that the excessive activity of the E7K mutant enough large to produce conduction block would result from its greatly facilitated C-O gating (Figure 5, Figure 6 and Figure 7) rather than its moderately increased expression [11].

The channel density (maximal activity)-dependent CV reduction was reported by a previous simulation study which adopted a different mathematical formulation to describe the wild-type TRPM4 channel gating [18]. However, there is a major difference between this and our studies in whether overexpression of wild-type TRPM4 can cause conduction block. While the former study demonstrated conduction failure with increased wild-type TRPM4 channel activity, our results show only negligible effects (Figure 6C and Figure 7A). There are at least three factors that could account for this discrepancy. First, Gaur et al. formulated voltage- and Ca^2+^-dependent gating of the wild-type TRPM4 channel as independent processes [18], but our model derived more complex formulations for TRPM4 channel gating by treating voltage and Ca^2+^ dependencies as inter-dependent processes. Accordingly, the extent of wild-type TRPM4 channel activation in our model becomes much weaker near RMP and resting [Ca^2+^]_i_ (which is, however, disrupted in the E7K mutant), as compared with the previous models which rather exaggerate the gating of TRPM4 or that of its native counterpart NSC_Ca_ around RMP [13,18]. Secondly, the Pan-Rudy model adopted by the previous study [18,21] appears more sensitive to increased TRPM4 channel activity than the human Purkinje fiber Trovato2020 model adopted in the present study [15]. In our early simulations using the Pan-Rudy model, we noticed that increasing TRPM4 channel density destabilizes the model to generate frequent forward/backward spontaneous AP propagations along the cable. This made the exact evaluation of conduction velocity difficult. Thirdly, the maximal activity or density of TRPM4 channels defined in the present study may be low (it is set to be twice as large as that of atrial myocytes; see the methods), although we referred to the fact that expression of this channel is at least a few fold higher in Purkinje fiber than in the human atrium [11]. However, doubling the maximal density merely shifts the CV density curves for E7K (Figure 6C and Figure 7A) to the left with least changes in that of the wild-type (Figure S2), and this makes the impact of E7K mutation even more prominent. Therefore, even though the assumptions made for our present simulations are not perfectly realistic, the conclusions discussed above will still hold valid.

Introducing heterogeneity in TRPM4 channel density in the cable produces not only monotonic decrease in conduction velocity but also partial blocks that split into non-conductive and full- or sub-conductive states (Figure 7B). This phenomenon was already reported by the preceding simulation work which observed more complex patterns of conduction failure classified as the first- to third-degree blocks [18]. Indeed, in our simulations as well, various types of partial blocks are observed when the linear gradient exists in the channel density along the cable or fibroblasts are inter-placed in the cable (Figure S3). The mechanism (s) underlying these complex phenomena remains entirely unclear but might involve the bifurcative properties of the model adopted and/or mutant TRPM4 channel gating per se [22]. In real settings, spatial distribution of proteins would not be homogenous even within the same tissues [23]. Thus, intercellular variations in transcriptional levels could introduce further complexities into conduction disorders in concert with inherited arrhythmogenicity.

In summary, the present study has disclosed a new pathogenic mechanism by which the E7K mutation induces brady-arrhythmogenicity, by use of the Iono-C/A recording-based gating analysis and numerical simulations with the most updated human Purkinje fiber model. The obtained results clearly show that at the single-cell level this mutation induces an excessive TRPM4 channel activity via acceleration and deceleration of its opening and closing transitions, respectively, but that the same alteration of gating simultaneously produces a variety of AP conduction blocks in cable-like multicellular arrangements. Further work will be needed to explore the exact clinical significance of these theoretical observations in future.

## 4. Materials and Methods

### 4.1. Cell Culture and Gene Transfection

Human embryonic kidney cells 293 (HEK293) were purchased from ATCC (Manassas, VA, USA) and maintained in Dulbecco’s modified Eagle medium supplemented with 10% fetal bovine albumin and a mixture of penicillin/streptomycin in a 100%-humidified, 5%CO_2_-gassed incubator at 35–36 °C, and were passaged every 3–4 days (up to 10–15 times). When reaching 70–90% confluency, HEK293 cells were dispersed by short trypsin treatment and gentle pipetting, and re-plated on cover slips pre-coated with poly L-lysin (30 μg/mL) for transfection. About 12 h later, the coverslips were incubated in a special medium (either DMEM or Opti-MEM^TM^, Gibco Life Technologies, Carlsbad, CA, USA) containing 1 μg pcI-neo vector encoding the CDS of *htrpm4b* gene or that of E7K mutant, with the aid of transfection agents superfect (Qiagen, Hilden, Germany) or lipofectatmine2000 ^TM^ (Invitrogen, Waltham, MA, USA) according to the manufactures’ instructions. Thirty-six to forty-eight hours after the transfection, electrophysiological recordings were carried out at room temperature. The human *trpm4b* cDNA (Gene ACC. No: AF497623) inserted in the pcDNA4TO-Flag vector was kindly provided by Profs. J.-P. Kinet (Beth Israel Deaconess Medical Center and Harvard Medical School, Boston, MA, USA) and P. Launay (INSERM, Paris, France). For experimental use, this was subcloned into the pcI-neo vector.

### 4.2. Electrophysiology

The whole-cell and cell-attached (C/A) variants of the patch-clamp technique were applied. Patch electrodes were fabricated from 1.5 mm borosilicate glass capillaries (Sutter Instrument), and joined to the headstage of a low noise, high impedance patch clamp amplifier (EPC10, HEKA Elektronik, Ludwigshafen, Germany). When filled with the internal solution, the input resistance of the electrodes ranged between 4–7 MΩ. An automated multi-channel data acquisition software ‘Patchmaster’ (HEKA, Germany) was used to control a patch amplifier, and >60% of series resistance was electronically offset. For long-term recordings, current and voltage signals were sampled by the Power Lab data acquisition system (AD Instruments, Sydney, Australia), and obtained data were analyzed offline.

The details of ionomycin-perforated cell-attached (Iono-C/A) recording were described in our former study [13]. Briefly, after ‘giga’ seal formation, the cell was quickly exposed to 5μM ionomycin-containing Ca^2+^-free, high K^+^ external solution, and then sequentially exposed to various extracellular concentrations ([Ca^2+^]_o_) (0.3, 1 and 5 mM). To null the resting membrane potential, ionomycin and Ca^2+^ were administered in the presence of high K^+^ throughout.

Single channel activities were recorded by the Iono-C/A method at 2 kHz digitization after 1 kHz low-pass filtering. The obtained data with no simultaneous multiple openings were re-filtered at 200 Hz (8-pole Bessel; rise time:1.66 ms) and subjected to dwell-time analysis. Dwell-time histograms were constructed after 2 ms binning and fitted by a sum of two exponentials, where events faster than 2 ms were ignored.

Power spectrum analysis was performed for the steady segments of macroscopic TRPM4 currents which were sampled at 1 kHz after 500 Hz low-pass filtering. The obtained data were re-filtered through 8-pole Butterworth at 200 Hz, from which power spectral density was calculated. The relationship between spectral density and frequency was then fitted to the equation: s(f) = s(0)/[1 + (f/f_c_)^2^], where s(f), s(0) and f_c_ denote spectral density at a frequency of interest (in Hz), that of 0 Hz, and corner frequency, respectively. 

For data analyses and illustrations, commercial software such as Clampfit v.10 (Axon Instruments, Foster City, CA, USA), Excel 2016 (Microsoft Office) and KaleidaGraph v.4 (Hulinks, Tokyo, Japan) were employed.

### 4.3. Solution

Standard external solution used for patch clamp experiments consisted of (in mM): 140 NaCl, 5 KCl, 1.2 MgCl_2_, 1.8 CaCl_2_, 10 Hepes, 10 glucose (adjusted to pH 7.4 with Tris base). High-K^+^ external solution for the Iono-C/A recording (in mM): 145 KCl, 1.2 MgCl_2_, 0, 0.3, 1 or 5 CaCl_2_, 10 Hepes, 10 glucose (adjusted to pH 7.4 with Tris base); pipette solution for the Iono-C/A recording: the standard external solution with 1 mM tetraethylammonium (TEA) and 100μM 4,4’-Diisothiocyano-2,2’-stilbenedisulfonic acid (DIDS) to block K and Cl channels. Ca^2+^ concentration of pipette solution was calculated by using a self-written program following Fabiato & Fabiato’s algorithm with the enthalpic and ionic strength corrections of association constants [24]. All test solutions were applied via a hand-made local perfusion system controlled by electrically driven solenoid valves (time for completing solution change: ~1 s).

### 4.4. Numerical Model Simulation

For single-cell action potential (AP) simulation, the code of a most updated human Purkinje fiber model [15] was downloaded from the CELLML repository [25], and run by Cor1.1 or OpenCOR [26]. The downloaded code was corrected for unit definition inconsistency. In order to incorporate the gating kinetics of TRPM4 channel and reproduce the time course of AP of the original Trovato 2020 model as exactly as possible, 40% of background Na conductance (I_Nab_) in the original model was replaced by TRPM4 channel conductance [defined as the permeability (P_Na_, P_K_) of 7.02 × 10^−8^ Litre/Farad/ms in Hodgkin-Huxley-Katz formalism] together with 5% increase and 5% decrease in the maximal conductances of I_Kr_ and I_to_, respectively. The values of P_Na_ and P_K_ chosen for TRPM4 are twice as large as those electrophysiologically determined for HL-1 atrial cardiomyocyte model [13,27,28], and this reflects the fact that expression level of TRPM4 protein is at least a few-fold higher in the Purkinje fiber than the atrium [11]. A very high correlation (0.9999) was obtained by these alterations between the original and modified models (Figure 6A). Corresponding changes in the other membrane conductance and intracellular Ca^2+^ dynamics are illustrated in Figure S4, where except for a newly added TRPM4 current and concomitant reduction in I_Nab_, differences between before and after the modification are only marginal. All models used were stabilized by at least 1000 runs after any modifications. As for the other single-cell AP models, we adopted modified Luo-Rudy 2000 ventricular AP model and Pan-Rudy 2011 Purkinje fiber model [21,29]. The 4th-order Runge-Kutta algorithm was used to solve ordinary differential equations in these models. Action potentials and membrane currents were iterated at a 0.001 ms interval. For illustrative purposes, computed results were output in 1 ms resolution.

For 1D-cable simulations, the ‘Chaste’ simulation package [30] was employed with a 3 cm-long cable discretized at every 0.01 cm (i.e., 301 nodes or 300 inter-nodal spaces; based on the data from [31]). A multi-core (Intel^®^ Core™ i7-6800K CPU @ 3.40 GHz × 12, Intel Corporation, Santa Clara, CA, USA), 64-bit parallel computer (FRGBX911/A, Frontier, Tokyo, Japan; OS: Ubuntu 14.04 LTS, Canonical Ltd., London, UK) was used to speed up the simulation. Since there are considerable variations in reported values [32,33], intracellular conductivity was set to 1.7 mS/cm or its five times larger value 8.5 mS/cm with the extracellular/intracellular conductivity ratio of 1.0 [32,33]. It should be mentioned, however, that increasing this ratio or the value of intracellular conductivity several-fold, or adopting a longer cable (4.8 cm) with a twice larger inter-nodal distance of 0.02 cm, albeit small quantitative differences, did not essentially affect the conclusions from the simulations (not shown). To attain steady AP conduction, simulations were performed for 25 s with time steps of 0.01 or 0.005 ms and 0.01 or 0.05 ms for ordinary and partial differential equations, respectively (solved by forward Euler and finite element methods, respectively), and the results were output at 0.1 ms resolution as a binary file containing all voltage data at every node and timepoint. These data were used to calculate conduction velocity along the cable and maximal upstroke slope of propagated AP and visualize the spatiotemporal profile of AP propagation as surface plots by self-written programs in Python3 (Python Software Foundation, Beaverton, OR, USA) or Matlab2021a (Mathworks, Natick, MA, USA). The results of analysis were illustrated by KaleidaGraph v.4 (Hulinks, Tokyo, Japan) or Excel 2016 (Microsoft, Redmond, WA, USA). 

### 4.5. Statistical Evaluation

All experimental data in Figure 1, Figure 2, Figure 3, Figure 4 and Figure 5 are expressed as the mean ± s.e.m. Statistical analysis was performed by the Student *t*-test. 

## Data Availability

The data described in this paper are available on request.

## Supplementary Materials

The following are available online at https://www.mdpi.com/article/10.3390/ijms22168513/s1.

## References

[1] Nilius B., Szallasi A. (2014). Transient receptor potential channels as drug targets: From the science of basic research to the art of medicine. Pharmacol. Rev..

[2] Launay P., Cheng H., Srivatsan S., Penner R., Fleig A., Kinet J.-P. (2004). TRPM4 regulates calcium oscillations after T cell activation. Science.

[3] Feng J., Zong P., Yan J., Yue Z., Li X., Smith C., Ai X., Yue L. (2021). Upregulation of transient receptor potential melastatin 4 (TRPM4) in ventricular fibroblasts from heart failure patients. Pflügers Arch.-Eur. J. Physiol..

[4] Hof T., Sallé L., Coulbault L., Richer R., Alexandre J., Rouet R., Manrique A., Guinamard R. (2016). TRPM4 non-selective cation channels influence action potentials in rabbit Purkinje fibres. J. Physiol..

[5] Hof T., Simard C., Rouet R., Sallé L., Guinamard R. (2013). Implication of the TRPM4 nonselective cation channel in mammalian sinus rhythm. Heart Rhythm.

[6] Mathar I., Kecskes M., Van der Mieren G., Jacobs G., Camacho Londoño J.E., Uhl S., Flockerzi V., Voets T., Freichel M., Nilius B. (2014). Increased β-adrenergic inotropy in ventricular myocardium from Trpm4−/− mice. Circ. Res..

[7] Pironet A., Syam N., Vandewiele F., Van den Haute C., Kerselaers S., Pinto S., Vande Velde G., Gijsbers R., Vennekens R. (2019). AAV9-mediated overexpression of TRPM4 increases the incidence of stress-induced ventricular arrhythmias in mice. Front. Physiol..

[8] Gueffier M., Zintz J., Lambert K., Finan A., Aimond F., Chakouri N., Hédon C., Granier M., Launay P., Thireau J. (2017). The TRPM4 channel is functionally important for the beneficial cardiac remodeling induced by endurance training. J. Muscle Res. Cell Motil..

[9] Kecskés M., Jacobs G., Kerselaers S., Syam N., Menigoz A., Vangheluwe P., Freichel M., Flockerzi V., Voets T., Vennekens R. (2015). The Ca^2+^-activated cation channel TRPM4 is a negative regulator of angiotensin II-induced cardiac hypertrophy. Basic Res. Cardiol..

[10] Vennekens R. (2018). Recent insights on the role of TRP channels in cardiac muscle. Curr. Opin. Physiol..

[11] Kruse M., Schulze-Bahr E., Corfield V., Beckmann A., Stallmeyer B., Kurtbay G., Ohmert I., Schulze-Bahr E., Brink P., Pongs O. (2009). Impaired endocytosis of the ion channel TRPM4 is associated with human progressive familial heart block type I. J. Clin. Investig..

[12] Rezazadeh S., Duff H.J. (2017). Genetic determinants of hereditary bradyarrhythmias: A contemporary review of a diverse group of disorders. Can. J. Cardiol..

[13] Hu Y., Duan Y., Takeuchi A., Hai-Kurahara L., Ichikawa J., Hiraishi K., Numata T., Ohara H., Iribe G., Nakaya M. (2017). Uncovering the arrhythmogenic potential of TRPM4 activation in atrial-derived HL-1 cells using novel recording and numerical approaches. Cardiovasc. Res..

[14] Hu Y., Li Q., Kurahara L.-H., Shioi N., Hiraishi K., Fujita T., Zhu X., Inoue R. (2021). An Arrhythmic Mutation E7K Facilitates TRPM4 Channel Activation via Enhanced PIP2 Interaction. Cells.

[15] Trovato C., Passini E., Nagy N., Varró A., Abi-Gerges N., Severi S., Rodriguez B. (2020). Human Purkinje in silico model enables mechanistic investigations into automaticity and pro-arrhythmic abnormalities. J. Mol. Cell. Cardiol..

[16] Nilius B., Mahieu F., Prenen J., Janssens A., Owsianik G., Vennekens R., Voets T. (2006). The Ca^2+^-activated cation channel TRPM4 is regulated by phosphatidylinositol 4, 5-biphosphate. EMBO J..

[17] Nilius B., Talavera K., Owsianik G., Prenen J., Droogmans G., Voets T. (2005). Gating of TRP channels: A voltage connection?. J. Physiol..

[18] Gaur N., Hof T., Haissaguerre M., Vigmond E.J. (2019). Propagation failure by TRPM4 overexpression. Biophys. J..

[19] Weidmann S. (1955). The effect of the cardiac membrane potential on the rapid availability of the sodium-carrying system. J. Physiol..

[20] Kléber A.G. (2005). The shape of the electrical action-potential upstroke: A new aspect from optical measurements on the surface of the heart. Circ. Res..

[21] Li P., Rudy Y. (2011). A model of canine purkinje cell electrophysiology and Ca^2+^ cycling: Rate dependence, triggered activity, and comparison to ventricular myocytes. Circ. Res..

[22] Prescott S.A., De Koninck Y., Sejnowski T.J. (2008). Biophysical basis for three distinct dynamical mechanisms of action potential initiation. PLoS Comput. Biol..

[23] Skelly D.A., Squiers G.T., McLellan M.A., Bolisetty M.T., Robson P., Rosenthal N.A., Pinto A.R. (2018). Single-cell transcriptional profiling reveals cellular diversity and intercommunication in the mouse heart. Cell Rep..

[24] Inoue R., Ito Y. (2000). Intracellular ATP slows time-dependent decline of muscarinic cation current in guinea pig ileal smooth muscle. Am. J. Physiol.-Cell Physiol..

[25] A Computational Human Cardiac Purkinje Electrophysiological Model. https://models.physiomeproject.org/e/5f0/ModTrovato2020.cellml/view.

[26] http://cor.physiol.ox.ac.uk/.

[27] Hu Y., Kaschitza D.R., Essers M., Arullampalam P., Fujita T., Abriel H., Inoue R. (2021). Pathological activation of CaMKII induces arrhythmogenicity through TRPM4 overactivation. Pflügers Arch.-Eur. J. Physiol..

[28] Takeuchi A., Kim B., Matsuoka S. (2013). The mitochondrial Na^+^-Ca^2+^ exchanger, NCLX, regulates automaticity of HL-1 cardiomyocytes. Sci. Rep..

[29] Luo C.-H., Rudy Y. (1994). A dynamic model of the cardiac ventricular action potential. II. Afterdepolarizations, triggered activity, and potentiation. Circ. Res..

[30] Chaste. http://www.cs.ox.ac.uk/chaste/.

[31] Callewaert G., Carmeliet E., Vereecke J. (1984). Single cardiac Purkinje cells: General electrophysiology and voltage-clamp analysis of the pace-maker current. J. Physiol..

[32] Clayton R., Bernus O., Cherry E., Dierckx H., Fenton F.H., Mirabella L., Panfilov A.V., Sachse F.B., Seemann G., Zhang H. (2011). Models of cardiac tissue electrophysiology: Progress, challenges and open questions. Prog. Biophys. Mol. Biol..

[33] Stinstra J.G., Hopenfeld B., MacLeod R.S. (2005). On the passive cardiac conductivity. Ann. Biomed. Eng..

