# Supplementary material for: Theoretical Investigation of the Mechanism by which A Gain-of-Function Mutation of the TRPM4 Channel Causes Conduction Block
Source: Int J Mol Sci. 2021 Aug 7;22(16):8513. doi: 10.3390/ijms22168513 (PMC8395173; doi:10.3390/ijms22168513)
Supplement: Supplementary file 1 [file ijms-22-08513-s001.zip › ijms-1293485-supplementary.pdf]

Supplementary Figures

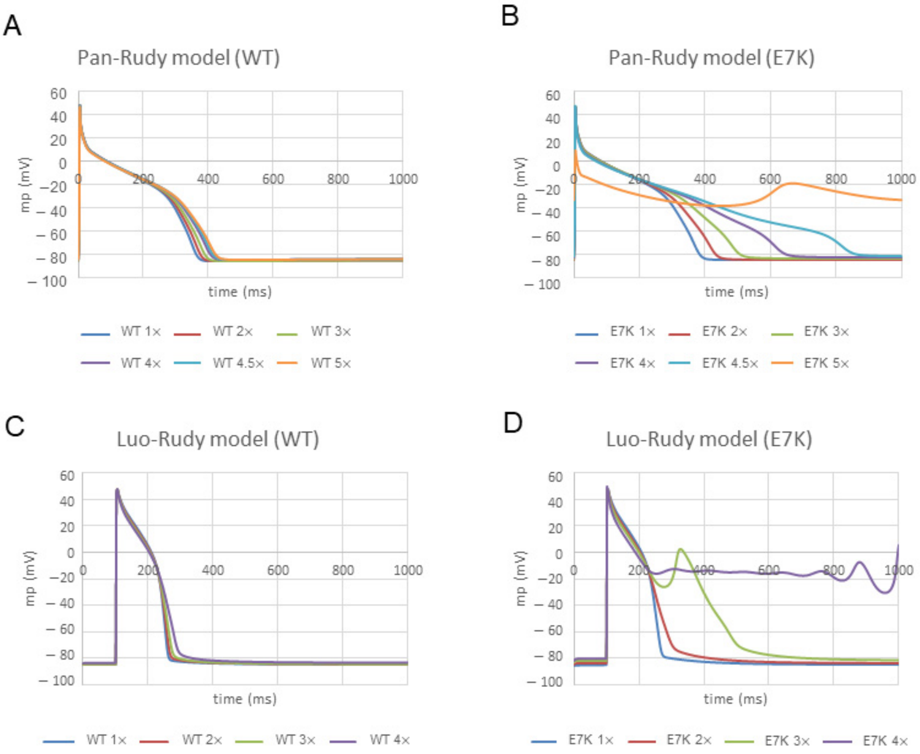

Figure S1: Effects of channel density increase on AP duration and shape.

A and B; overlaid traces of APs simulated by modified Trovato 2020 model with the gating kinetics of either wild-type (A) or E7K mutant (B) TRPM4 channel. The channel density is presented as a multiple (×) of control (i.e. 7.02×10^{-8} Litre/farad/ms). C and D; overlaid traces of APs simulated by modified Luo-Rudy 2000 model with the gating kinetics of either wild-type (C) or E7K mutant (D) TRPM4 channel. The channel density is presented as a multiple (×) of the pre-set value in the original Luo-Rudy model (i.e. 3.51×10^{-7} Litre/farad/ms).

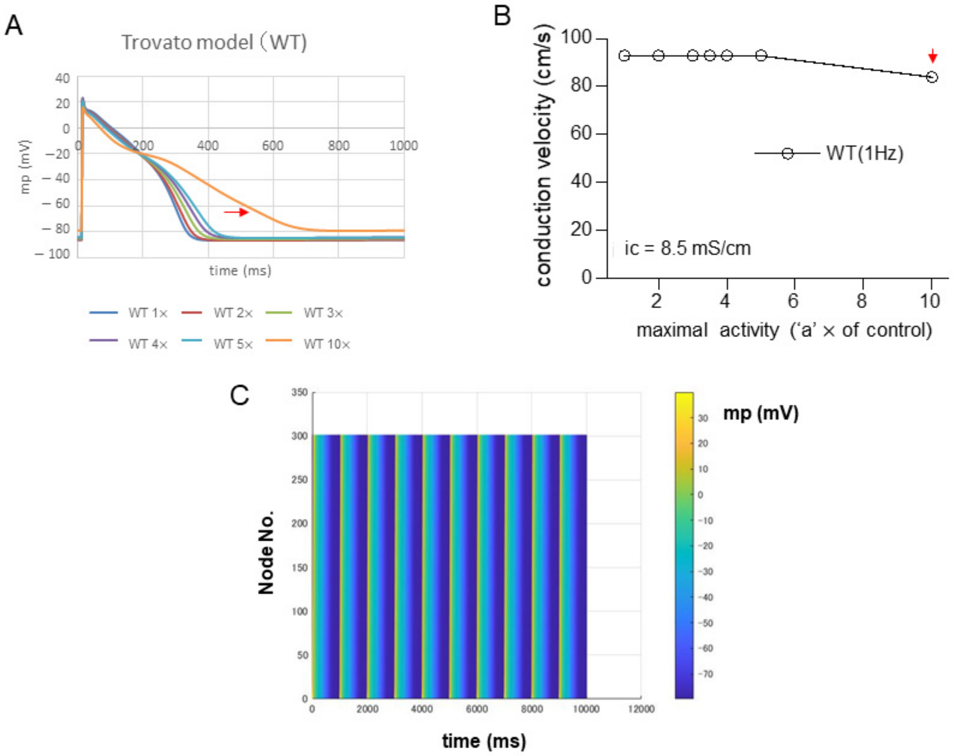

Figure S2: 10 times increase in channel density only slightly affects AP conduction.

The graphs in Figs.6B (left) and 7A are extended to include 10-fold (10x) increase in the density (or maximal activity) of wild-type TRPM4 channel. A; overlaid traces of APs simulated by modified Trovato model. B; relationship between channel density and conduction velocity (CV) calculated based on the results of 1D-cable simulation with modified Trovato model. The same conditions as in Figs.6 and are used. C; spatiotemporal profile of AP propagation (last 10 stimulations) for 10-fold increase in wild-type TRPM4 channel density. Although there is considerable prolongation of AP duration by this increase (A), the resultant reduction in CV is only about 10% (B).

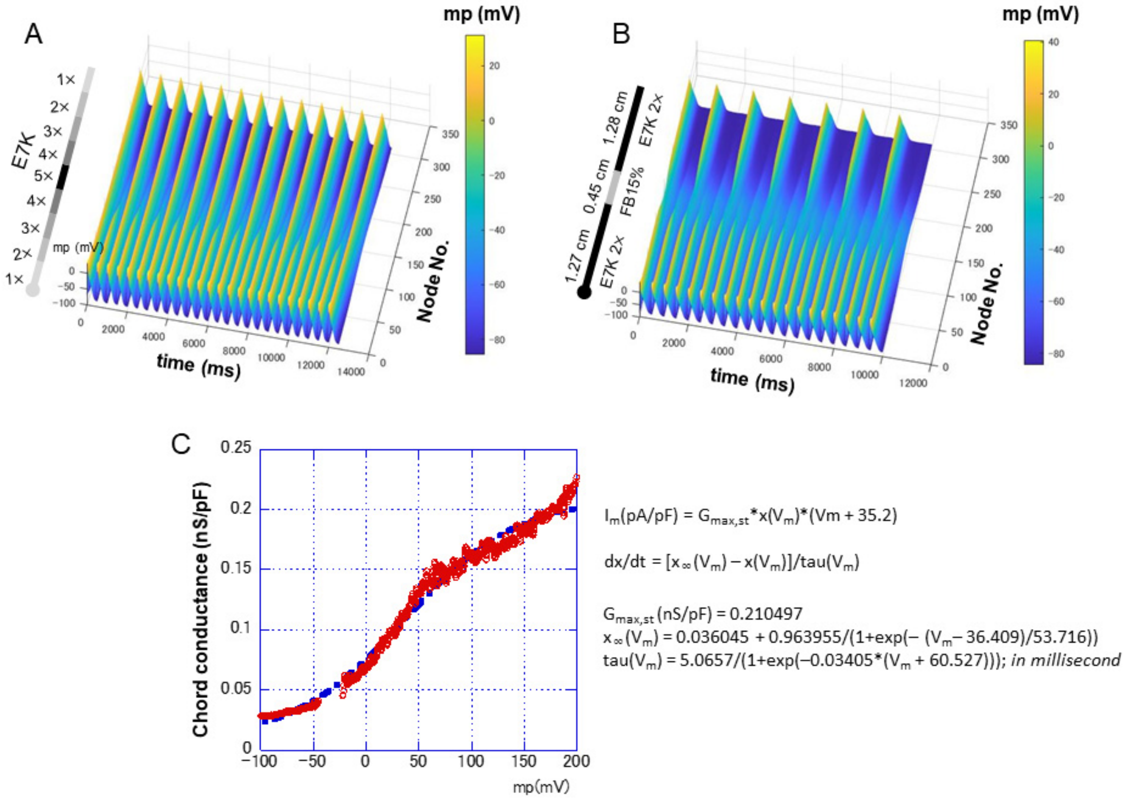

Figure S3: Heterogeneity in channel density or cell type causes various degrees of conduction blocks. A and B; spatiotemporal profiles of AP propagation (2:1 block) derived from 1D-cable simulation (2Hz pacing) with modified Trovato model for the cable with a triangular gradient of channel density (1x - 5x) (A), and that (3:1 block) with 15% replacement by cardiac fibroblasts (FB) in the middle region (B). Mathematical formulation for FB electrophysiology was made based on the data shown in C. C; averaged chord conductance-voltage relationship of cardiac fibroblasts obtained by patch clamp experiments (n=5), which is mathematically formulated with time constants of voltage-dependent activation that were determined separately.

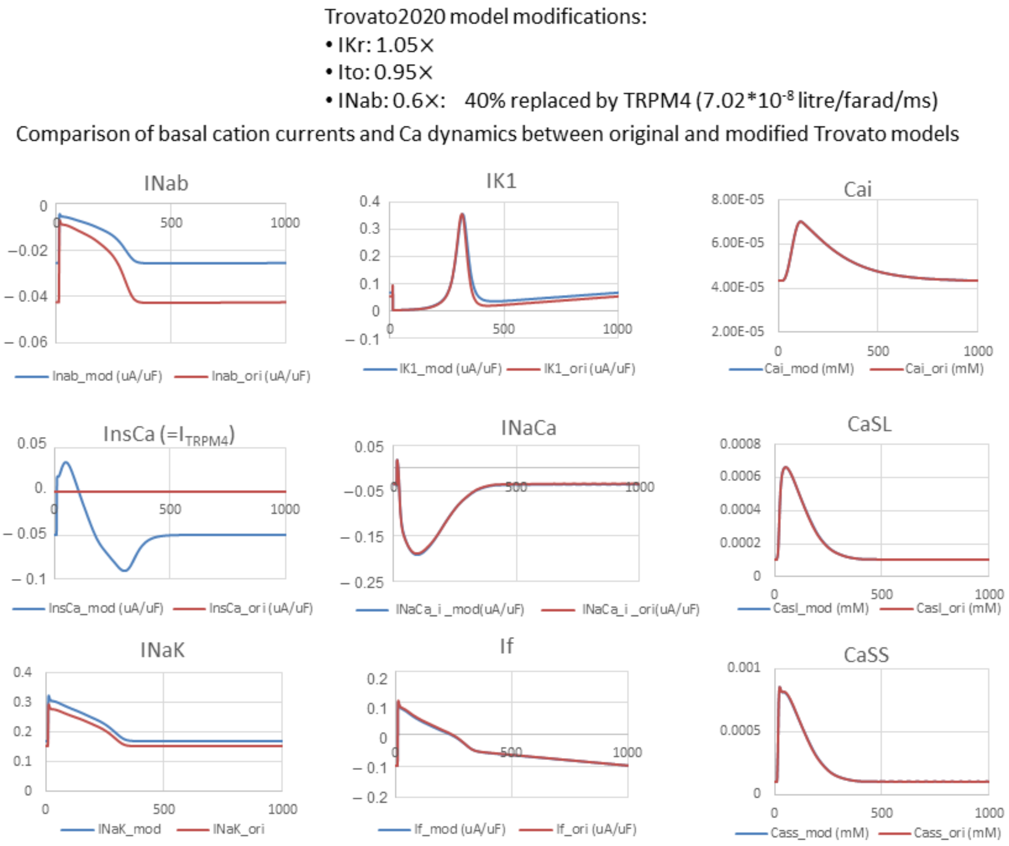

Figure S4: Adaptation of TRPM4 gating to Trovato 2020 model.

Overlaid traces for simulated basal cation currents and intracellular Ca^{2+} concentrations before (*.ori, red) and after (*.mod, blue) adaptation of TRPM4 gating. From top to bottom; left column: background Na^+ current (INab), Ca^{2+}-activate nonselective cation current (InsCa or TRPM4-mediated current), Na^+/K^+ pump current (INaK); middle column: inward rectifying K+ current (IK1), Na^+/Ca^{2+} exchanger current (INaCa), hyperpolarization-activated cation current (If); right column: Cai, CaSL, CaSS. Notations follows Trovato et al (2020).
